# Effects of Taekwondo training on thigh muscle cross-sectional area, health-related physical fitness, HbA1c, and GLP-1 in sedentary older women

**DOI:** 10.3389/fspor.2025.1553202

**Published:** 2025-04-03

**Authors:** Jaehyun Park, Bongjo Kim, Minki Jeong, Hyun-Hun Jung, Garam Hong, Sang Kab Park

**Affiliations:** ^1^ College of General Education, Tongmyong University, Busan, Republic of Korea; ^2^ Department of Physiology, Dong-A University College of Medicine, Busan, Republic of Korea; ^3^ College of Arts and Sports, Dong-A University, Busan, Republic of Korea; ^4^ Waseda Institute for Sport Sciences, Waseda University, Saitama, Japan

**Keywords:** Taekwondo, thigh muscle cross-sectional area, health-related physical fitness, metabolic syndrome, insulin resistance, older women

## Abstract

**Background:**

Sedentary lifestyles in older individuals are associated with reduced physical function and an increased risk of metabolic diseases such as type 2 diabetes. Physical exercise can enhance muscle mass, insulin sensitivity, and metabolic health. Taekwondo, a martial art that integrates both aerobic and resistance components, may improve strength, balance, and metabolic health in older individuals. This study investigated the effect of long-term Taekwondo training on thigh muscle cross-sectional area, health related physical fitness, and metabolic indicators in sedentary older women.

**Methods:**

Seventeen participants (aged 65 years and older, sedentary time 8 h and more per day) were randomly assigned to a Taekwondo group (*n* = 9) and a control group (*n* = 8). Outcomes, including thigh muscle cross-sectional area, health-related physical fitness, Homeostatic Model Assessment for Insulin Resistance (HOMA-IR), and Glucagon-like peptide-1 (GLP-1) were measured before and after the Taekwondo program. The Taekwondo group underwent 60 min of training, three times per week for 12 weeks. Variable changes over time and between groups were analyzed using two-way repeated measures ANOVA performed for significant interactions.

**Results:**

The Taekwondo group exhibited a significant reduction in body weight, body mass index, body fat, and mean arterial blood pressure (*p* < 0.05), as well as increased thigh muscle cross-sectional area, lean body mass and lower limb muscle mass (*p* < 0.05). Improvements in balance and gait speed, stride were observed (*p* < 0.05), indicating reduced fall risk and enhanced mobility. Laboratory analyses revealed reduced triglyceride and free fatty acids and elevated HDL-cholesterol and GLP-1 levels (*p* < 0.05). Increased thigh muscle cross-sectional area was inversely correlated with fasting glucose, insulin, and HOMA-IR, suggesting improved insulin sensitivity and glucose regulation.

**Conclusion:**

Long-term Taekwondo training improved thigh muscle cross-sectional area, health-related physical fitness and insulin resistance markers in sedentary older women, providing evidence for its use as an effective intervention to promote metabolic health in this population.

## 1 Introduction

The global average life expectancy was 73.6 years in 2022 and is projected to increase to 78.1 years by 2050 (1). However, as life expectancy increases, the health of the older individual population has emerged as a significant social issue and is recognized as a primary factor contributing to a decline in quality of life (2).

A sedentary lifestyle has a deleterious impact on the general health of older individuals (3, 4). Recent studies have also demonstrated that sedentary behavior negatively impacts thigh muscle cross-sectional area (CSA), leading to accelerated muscle atrophy and reduced functional capacity (5, 6). This is also, associated with an increased risk of metabolism-related conditions such as insulin resistance, metabolic syndrome, and type 2 diabetes, musculoskeletal conditions such as knee pain, sarcopenia and osteoporosis (7–9). In contrast, individuals who engage in reduced sedentary behavior, light physical activities, or simple muscle-strengthening exercises have been reported to exhibit superior health outcomes (10–13). More importantly, exercise can optimize these effects, which is crucial in reducing insulin resistance and preventing metabolic syndrome in older individuals (14, 15). Addressing these issues through targeted exercise interventions is essential for maintaining independence and overall well-being in aging populations.

Aerobic and anaerobic exercise have been independently demonstrated to enhance insulin sensitivity and facilitate glucose homeostasis, contributing to improved glycated hemoglobin (HbA1c) levels (16–18). Furthermore, exercise-induced increases in glucagon-like peptide-1 (GLP-1) levels play a significant role in improving insulin resistance (19). These changes have beneficial effects on muscle mass and insulin resistance, which contribute to overall metabolic health.

Thigh muscle mass is a crucial indicator of physical function and health status in older individuals, as it is directly associated with overall health (20). Thigh muscles comprise approximately 50%–80% of total body muscle mass and are essential for maintaining overall health, balance, and gait stability (21). In older adults, a reduction in thigh muscle mass is associated with an elevated risk of falls, diminished strength, increased prevalence of chronic disease, insulin resistance, and diminished quality of life (8, 22). Exercise that increases thigh muscle mass is effective in addressing these issues, thereby enhancing the overall physical function of older individuals (23).

Taekwondo, a widely accessible martial art in Korea, confers advantageous effects on physical function, and body composition in older individuals (24–27). As a combined aerobic and anaerobic exercise, Taekwondo, can contribute to improvements not only in strength and balance, but also cardiorespiratory endurance (24, 25, 28). The dynamic movements, including kicks, postural shifts, and coordinated footwork, provide significant muscle engagement that stimulates strength gains, particularly in the lower body (29).

Consequently, this can help prevent and improve the risk factors for sarcopenia (30), thus promoting the overall health of older individuals. The present study aims to assess the effects of regular and long-term Taekwondo training on thigh muscle CSA, health-related physical fitness, HbA1c, and GLP-1 levels in sedentary older women, providing valuable insights into the potential of martial arts as a multifaceted approach to healthy aging.

## 2 Materials and methods

### 2.1 Study participants

The participants were female residents aged 65 years or older from Busan city, Republic of Korea, who had no limitations in physical activity by a physician. A total of 26 participants were recruited and randomly assigned to either the Taekwondo training group (*n* = 13) or a control group (*n* = 13) using simple randomization via a computer-generated random sequence (https://www.ranmomizer.org).

Sedentary time was assessed using a wrist-worn PA monitor (Polar Active, Polar Electro, Finland), which participants were instructed to wear continuously for one week prior to the intervention. To classify sedentary behavior, an energy expenditure threshold of <1.5 METs was applied, following established guidelines for sedentary behavior assessment (31).

In the final analysis, 17 participants were included, while eight were excluded due to sedentary time non-eligibility (*n* = 2), relocation (*n* = 1), medical conditions (*n* = 1), attendance below 95% of the scheduled sessions (*n* = 4), and loss to follow-up (*n* = 1). Consequently, the analysis was conducted using data from 17 participants who fully adhered to the intervention and assessments, with 9 in the Taekwondo group and 8 in the control group. Participants who remained in the study attended at least 95% of the scheduled sessions. No serious injuries were reported during the intervention; however, some participants experienced minor discomfort, such as muscle soreness. Participants taking medication were instructed to maintain their usual lifestyle, including diet and physical activity habits, to ensure consistency. Baseline characteristics of the study participants are presented in Table 1.

**Table 1:** Baseline characteristics of subjects.

Variable	Taekwondo (*n* = 9)	Control (*n* = 8)	*p*-value
Age (years)	73.00 ± 5.68	71.88 ± 4.32	0.743
Body height (m)	1.49 ± 0.02	1.49 ± 0.06	0.963
Body mass (kg)	58.89 ± 6.68	54.95 ± 4.38	0.200
BMI (kg/m^2^)	26.54 ± 2.94	24.91 ± 2.25	0.236
LBM (kg)	33.31 ± 3.57	31.88 ± 3.09	0.370
Body fat (%)	43.27 ± 4.13	41.84 ± 5.49	0.541
Waist circumference (cm)	92.36 ± 6.68	90.05 ± 6.26	0.370
WHR	0.91 ± 0.04	0.94 ± 0.04	0.200
Lower limb muscle mass (kg)	9.69 ± 1.12	9.58 ± 1.14	0.673
ASM (kg)	13.17 ± 1.53	12.85 ± 1.42	0.606
MAP (mmHg)	106.56 ± 13.81	103.21 ± 9.59	0.370

Values are represented as mean ± standard deviation.

*p*-values were analyzed by Mann–Whitney *U* test.

BMI, body mass index; LBM, lean body mass; WHR, waist-hip ratio; LLM, lower limb muscle mass; ASM, appendicular skeletal muscle mass; MAP, mean arterial pressure.

This study was conducted in accordance with the principles of the Declaration of Helsinki and approved by the Institutional Review Board of D University (IRB No. 2-1040709-AB-N-01-201904-HR-022-04). Written informed consent was obtained from all participants.

### 2.2 Measurement of body composition

Body composition was evaluated using a body composition analyzer (VENUS 5.5, Jawon Medical Co., Korea) to ascertain height, weight, lean body mass (LBM), and percent body fat. Waist and hip circumferences were measured with a Martin-type measuring tape, and the waist-hip ratio (WHR) was calculated using the following formula: waist circumference divided by hip circumference. Blood pressure was determined using a mercury-free sphygmomanometer (CK-E301, Chin Kow Medical Instrument Co., Ltd., Taiwan), and mean arterial pressure (MAP) was calculated according to the formula MAP = diastolic blood pressure + [(systolic blood pressure − diastolic blood pressure)/3], as previously reported (32).

### 2.3 Thigh muscle cross-sectional area

The thigh muscle CSA was evaluated via computed tomography (CT) using a CT Max II scanner (General Electric Co., USA). The scan was conducted in a transverse plane at the umbilicus level, specifically crossing the midpoint between the upper border of the patella and the greater trochanter. The mass of the thigh muscles was defined by regions with Hounsfield numbers ranging from −49 to +100, as described by Choi et al. (33).

### 2.4 Health-related physical fitness

HRPF was assessed using a set of physical fitness tests, including hand grip strength (34), the 2-minute walk test (35), the open-eyed single-leg stance test (SLST) (36), the timed up-and-go (TUG) test (37), the 4-m gait speed test (38), maximal stride length (39), and the short physical performance battery (SPPB) (40).

### 2.5 Blood collection and laboratory assays

Blood samples were collected and analyzed under standardized procedures. To minimize any circadian rhythm variations, all blood samples were collected in the morning hours (between 08:00 and 11:00 h). Participants were instructed to fast for at least 10–12 h before sample collection. Upon arrival at the laboratory, 15 ml of venous blood was collected from the antecubital vein of each participant.

For the analysis of total cholesterol, triglyceride (TG), high-density lipoprotein cholesterol (HDL-C), low-density lipoprotein cholesterol (LDL-C), and fasting blood glucose (FBG) levels, blood was collected into tubes containing clotting activators for serum isolation. The samples were allowed to clot for 45 min at room temperature and centrifuged at 3,000 rpm for 10 min at 4 °C. After centrifugation, serum was dispensed into plain microtubes and stored at −80 °C until analysis. These biomarkers were measured using enzymatic methods (Cobas c702, Roche Diagnostics, Basel, Switzerland).

Insulin levels were quantified using an electrochemiluminescence immunoassay (Elecsys Insulin Kit, Roche Diagnostics, Basel, Switzerland). The homeostasis model assessment of insulin resistance (HOMA-IR) was calculated according to the formula [fasting insulin [μU/ml] × fasting plasma glucose [mg/dl]/405], as proposed by Matthews et al. (41). HbA1c levels were determined using an automated glycohemoglobin analyzer (HLC-723G8, Tosoh Corporation, Tokyo, Japan), and GLP-1 levels were assessed using enzyme-linked immunosorbent assays (ELISA Kit, R&D Systems, Minneapolis, MN, USA).

### 2.6 Taekwondo training program

The Taekwondo training program was adapted and modified from the recommendations set forth by Jung et al. (28) and was based on the American College of Sports Medicine's (ACSM) frequency, intensity, type, and time recommendations for older individuals (42–44). Each session consisted of a 10-min warm-up, 40-min main exercise, and 10 min cool-down, conducted three times per week for 12 weeks. The primary exercise consisted of fundamental Taekwondo techniques and kicking maneuvers executed with Thera-Bands for resistance training. In the initial 4-week period, the participants engaged in the practice of fundamental movements. During the subsequent 8-week phase, they performed Taekwondo movements with a yellow Thera-Band. In the final 4-week period, they utilized a red Thera-Band, progressively elevating the intensity of exercises. In accordance with the ACSM (45), the intensity of the exercise was increased at 4-week intervals, and the Rating of Perceived Exertion (RPE) was evaluated according to Borg (46). To ensure participant safety and adherence, adjustments were made when necessary, including modifying the range of motion or reducing the number of repetitions based on individual conditions. All modifications were implemented under the supervision of exercise professionals. The specifics of the Taekwondo training program are outlined in Table 2.

**Table 2:** Taekwondo training program.

Item	Contents			Time (min)
Warm-up	Shuttle walking, sit down and stretch forward, lean forward with your legs open, ankle twist, raise one leg up and lean forward (both sides), put your feet together and lean forward, put your hands together and lean back, clasped up with both hands, clasped both hands and stretches to the side, lean forward with both hands clasped, push both arms against the wall			10
Exercises	1–4 week	Seogi–Moaseogi, Naranhiseogi, Juchumseogi, Apseogi, Jireugi–Underneath, Trunk, Face opposite, Makgi–Naeryeomakgi, Anmakgi, Ollyeomakgi, Chagi(sitting)–Apchagi, Dollyeochagi, Naeryeochagi *Repeat 10–12 times after memorizing each action	RPE: <10	40
	5–8 week	Seogi–Moaseogi, Naranhiseogi, Juchumseogi Jireugi–Underneath, Trunk, Face opposite, Side Makgi–Naeryeomakgi, Anmakgi, Ollyeomakgi Chagi(sitting)–Apchagi, Dollyeochagi, Naeryeochagi *10 repetitions after memorizing each action (2set)	RPE: 10–11	
			Thera-Band Yellow	
	9–12 week	Seogi–Apgubi, Dwitgubi, Beomseogi Jireugi–Underneath, Trunk, Face opposite, Side Makgi–Yeommakgi, Bakkanmakgi, Biteureomakgi Chagi(standing)-Apchagi, Dollyeochagi, Naeryeochagi *10 repetitions after memorizing each action (3 set)	RPE: 12–13	
			Thera-Band Red	
Cool-down	Sit down and stretch forward, lean forward with your legs open, ankle twist, raise one leg up and lean forward (both sides), put your feet together and lean forward, put your hands together and lean back, clasped up with both hands, clasped both hands and stretches to the side, lean forward with both hands clasped, push both arms against the wall			10

### 2.7 Statistical analysis

All variables were analyzed using SPSS version 22.0 for Windows (IBM, Chicago, IL, USA). Descriptive statistics, including means and standard deviations, were calculated for all variables.

The homogeneity of baseline characteristics between the Taekwondo and control groups was assessed using either an independent *t*-test or the Mann–Whitney *U* test, depending on the data distribution. To evaluate between-group differences over time, a two-way repeated measures ANOVA was conducted. Spearman's rank correlation coefficient was employed to determine inter-variable relationships, and linear regression analysis was conducted to examine scatter plots. Statistical significance was set at *p* < 0.05.

## 3 Results

### 3.1 Thigh muscle cross-sectional area and body composition

As indicated in Table 3, the results of thigh muscle CSA and body composition are as follows. The Taekwondo group exhibited a significant reduction in body mass (*p* < 0.05), body mass index (BMI) (*p* < 0.01), percent body fat (*p* < 0.05), and MAP (*p* < 0.05) after 12 weeks. Conversely, there was a significant increase in thigh muscle CSA (*p* < 0.01), appendicular skeletal muscle mass (ASM) (*p* < 0.05). Furthermore, the interaction between-group and time was significant for thigh muscle CSA (*p* = 0.013), body mass (*p* = 0.007), BMI (*p* < 0.001), LBM (*p* = 0.013), percent body fat (*p* = 0.010), WHR (*p* = 0.040), LLM (*p* = 0.025), ASM (*p* = 0.008), and MAP (*p* = 0.015).

**Table 3:** Changes in thigh muscle cross-sectional area, body composition between groups at baseline and after 12 weeks.

Variable	Group	Baseline	12 weeks	% diff	*p*-value (interaction)
Thigh muscle CSA (mm^2^)	Taekwondo	8,913.44 ± 1,695.53	9,804.56 ± 1,331.31	10.00**	0.013
	Control	8,367.56 ± 1,138.79	8,305.33 ± 968.63	−1.32	
Body mass (kg)	Taekwondo	58.89 ± 6.68	57.54 ± 5.45	−2.28*	0.007
	Control	55.38 ± 4.29	55.80 ± 3.69	1.16	
BMI (kg/m^2^)	Taekwondo	26.54 ± 2.94	25.66 ± 2.46	−3.34**	<0.001
	Control	24.91 ± 2.25	25.14 ± 2.15	1.19	
LBM (kg)	Taekwondo	33.31 ± 3.57	34.71 ± 3.39	4.20*	0.013
	Control	31.92 ± 2.89	32.36 ± 2.50	1.88	
Percent body fat (%)	Taekwondo	42.27 ± 4.13	40.80 ± 4.28	−5.70*	0.010
	Control	42.19 ± 5.25	42.41 ± 4.88	0.48	
WHR	Taekwondo	0.91 ± 0.04	0.89 ± 0.03	−1.50	0.040
	Control	0.94 ± 0.03	0.95 ± 0.03	2.13	
LLM (kg)	Taekwondo	9.69 ± 1.12	9.88 ± 1.10	2.00	0.025
	Control	9.66 ± 1.09	9.41 ± 0.92	−2.26	
ASM (kg)	Taekwondo	13.17 ± 1.53	13.65 ± 1.36	3.64*	0.008
	Control	12.90 ± 1.34	12.61 ± 1.16	−1.96	
MAP (mmHg)	Taekwondo	106.56 ± 13.81	98.59 ± 7.06	−7.47*	0.015
	Control	105.30 ± 10.94	106.41 ± 7.47	2.58	

Values are represented as mean ± standard deviation.

Significantly different from baseline: *p < 0.05, **p < 0.01.

Thigh muscle CSA, thigh muscle cross-sectional area; BMI, body mass index; LBM, lean body mass; WHR, waist-hip ratio; LLM, lower limb muscle mass; ASM, appendicular skeletal muscle mass; MAP, mean arterial pressure.

**Figure 1:**
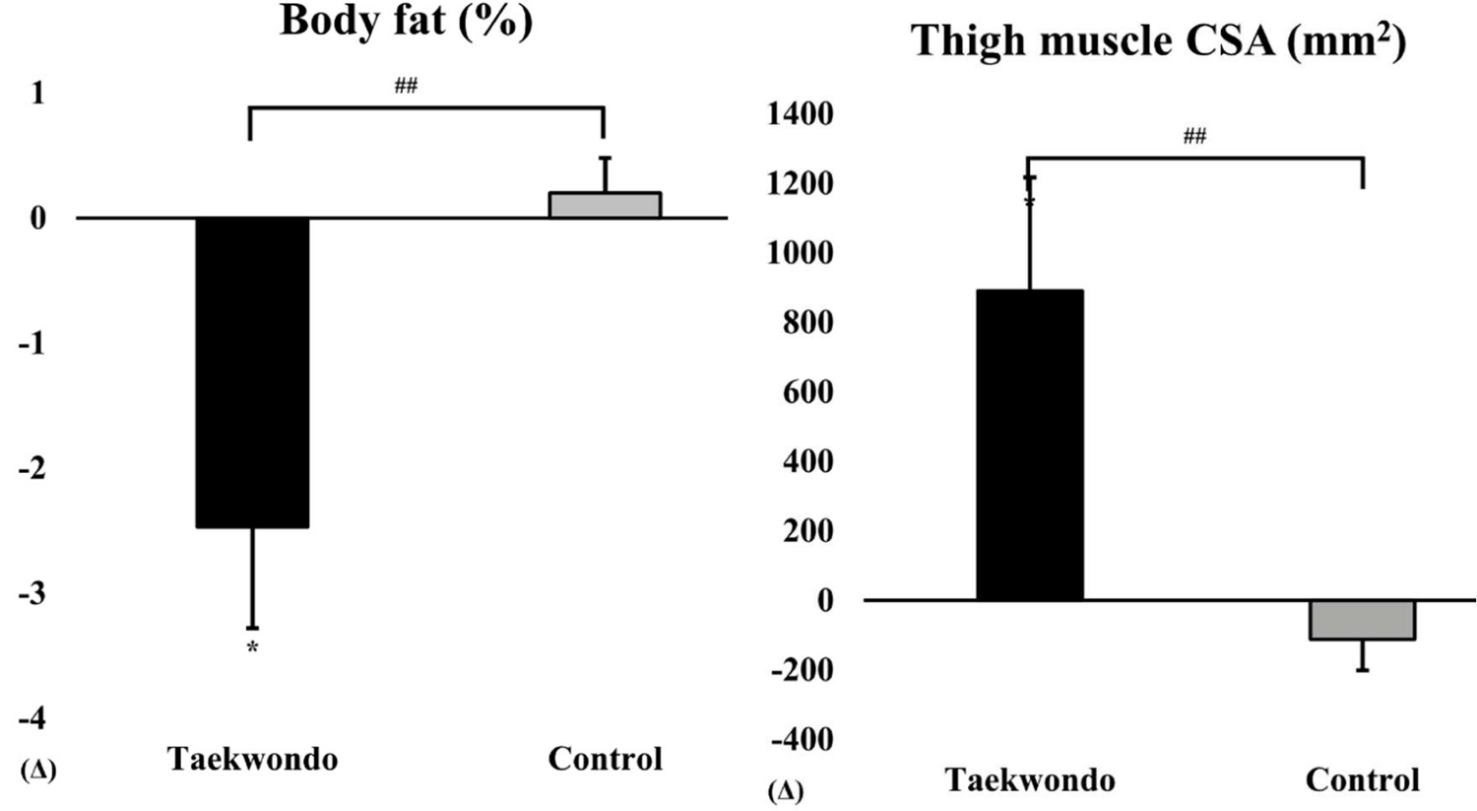
Comparison of body fat (%) and thigh muscle CSA between baseline and after 12 weeks in taekwondo program. #: *p*-value (interaction) ##*p* < 0.01. *: *p*-value (time) *p < 0.05. Δ: Post–Pre. Thigh muscle CSA, thigh muscle cross-sectional area.

As shown in Figure 1, the Taekwondo group exhibited a significant reduction in body fat percentage compared to baseline (*p* < 0.05), whereas the control group showed no significant changes. Additionally, thigh muscle CSA significantly increased in the Taekwondo group (*p* < 0.01), while the control group exhibited a non-significant decrease. Significant interaction effects were observed for both variables (*p* < 0.05).

### 3.2 Health-related physical fitness

HRPF and SPPB outcomes are presented in Table 4. In the Taekwondo group, significant post-intervention improvements were observed in the SLST (*p* < 0.01), maximal stride length (*p* < 0.05), and SPPB (*p* < 0.05). Additionally, the TUG test showed a significant reduction (*p* < 0.01).

**Table 4:** Changes in HRPF between groups at baseline and after 12 weeks.

Variable	Group	Baseline	12 weeks	% diff	*p*-value (interaction)
Hand grip strength (kg) (dominant)	Taekwondo	21.29 ± 3.95	22.09 ± 3.58	3.76	0.566
	Control	21.36 ± 2.86	21.40 ± 2.94	0.03	
Repeated chair stand (s)	Taekwondo	8.76 ± 2.21	7.95 ± 1.84	−9.34	0.012
	Control	8.77 ± 2.62	9.24 ± 2.51	6.20	
2-minute walking test (frequency)	Taekwondo	103.67 ± 26.33	110.89 ± 21.08	6.97	0.085
	Control	101.22 ± 24.83	92.78 ± 31.28	−8.51	
SLST(s) (dominant)	Taekwondo	4.02 ± 3.68	11.27 ± 6.40	180.35**	0.003
	Control	3.44 ± 1.98	2.83 ± 2.14	−16.88	
TUG (s)	Taekwondo	6.92 ± 0.65	6.33 ± 0.56	−8.54**	0.011
	Control	6.24 ± 0.71	6.61 ± 1.11	4.05	
Normal gait speed (m/s)	Taekwondo	1.01 ± 0.21	1.07 ± 0.17	5.21	0.403
	Control	0.99 ± 0.21	0.92 ± 0.11	−4.48	
Maximal gait speed (m/s)	Taekwondo	0.69 ± 0.06	0.70 ± 0.03	1.53	0.045
	Control	0.67 ± 0.07	0.61 ± 0.08	−9.47	
Maximal stride length (cm)	Taekwondo	78.78 ± 14.02	81.11 ± 12.98	2.96*	0.013
	Control	78.89 ± 13.40	70.78 ± 15.00	−6.92	
SPPB (score)	Taekwondo	10.22 ± 1.20	11.22 ± 1.56	9.78*	0.010
	Control	9.89 ± 1.36	9.56 ± 1.33	−3.75	

Values are represented as mean ± standard deviation.

Significantly different from baseline: *p < 0.05, **p < 0.01.

SLST, single-leg stance test; TUG, timed up and go; SPPB, short physical performance battery.

Moreover, significant interaction effects between-group and time were identified for repeated chair stand (*p* = 0.012), SLST (*p* = 0.003), TUG test (*p* = 0.011), maximal gait speed (*p* = 0.045), maximal stride length (*p* = 0.013), and SPPB (*p* = 0.010).

**Figure 2:**
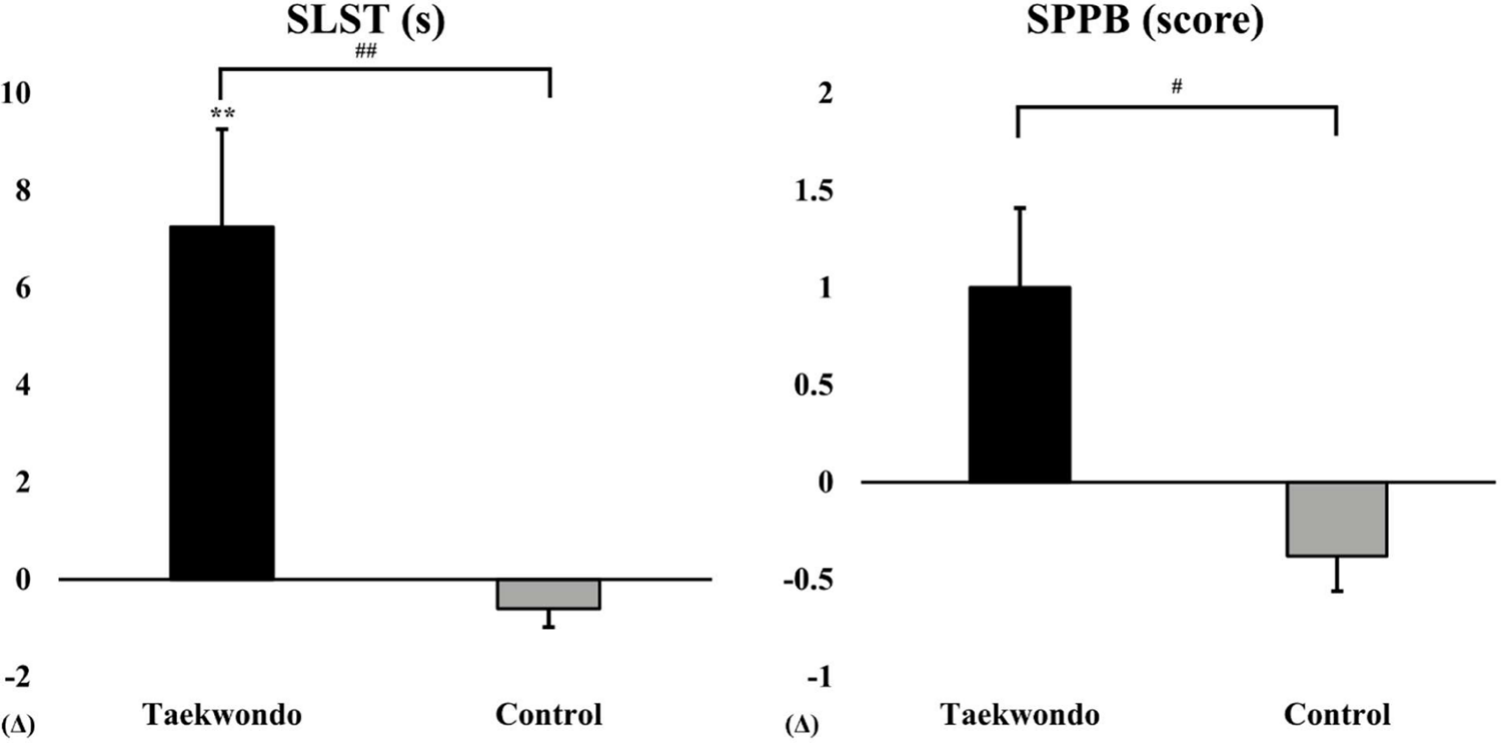
Comparison of SLST and SPPB between baseline and after 12 weeks in taekwondo program. #: *p*-value (interaction) #*p* < 0.05, ##*p* < 0.01. *: *p*-value (time) **p < 0.01. Δ: Post–Pre. SLST, single-leg stance test; SPPB, short physical performance battery.

As shown in Figure 2, the Taekwondo group exhibited a significant increase in SLST time compared to baseline (*p* < 0.01), whereas the control group showed no significant changes. Additionally, the SPPB score increased in the Taekwondo group, while the control group exhibited a slight decrease. Significant interaction effects were observed for SLST (*p* < 0.01) and SPPB (*p* < 0.05).

### 3.3 Laboratory markers

Changes in blood lipid levels and insulin resistance markers are presented in Table 5. In the Taekwondo group, significant reductions were observed in TG, FFA, FBG, insulin, HbA1c, and HOMA-IR whereas GLP-1 levels showed a significant increase. Moreover, significant interaction effects between-group and time were observed for TG (*p* = 0.036), HDL-C (*p* = 0.031), FFA (*p* = 0.045), FBG (*p* = 0.009), HbA1c (*p* = 0.025), and GLP-1 (*p* < 0.001) levels.

**Table 5:** Changes in laboratory markers.

Variable	Group	Baseline	12 weeks	% diff	*p*-value (interaction)
TC (mg/dl)	Taekwondo	173.67 ± 44.59	159.67 ± 13.16	−8.06	0.226
	Control	165.13 ± 34.65	178.75 ± 30.01	8.25	
TG (mg/dl)	Taekwondo	126.00 ± 43.30	92.78 ± 23.95	−26.37*	0.036
	Control	132.56 ± 48.13	144.33 ± 43.68	9.55	
HDL-C (mg/dl)	Taekwondo	56.44 ± 12.02	64.44 ± 11.64	14.17	0.031
	Control	60.00 ± 19.03	51.00 ± 6.28	−15.95	
LDL-C (mg/dl)	Taekwondo	106.44 ± 38.35	104.11 ± 13.51	−2.19	0.227
	Control	90.00 ± 31.24	103.88 ± 32.87	15.42	
FFA (μmol/L)	Taekwondo	606.78 ± 340.26	498.56 ± 222.32	−17.84*	0.045
	Control	608.13 ± 237.90	726.38 ± 250.77	6.80	
FBG (mg/dl)	Taekwondo	120.56 ± 13.70	100.44 ± 12.89	−16.68*	0.009
	Control	117.44 ± 8.95	120.56 ± 6.73	2.65	
Insulin (μU/ml)	Taekwondo	19.59 ± 16.69	8.24 ± 3.84	−57.91*	0.058
	Control	11.44 ± 7.00	13.09 ± 6.40	14.77	
HbA1c (%)	Taekwondo	5.93 ± 0.47	5.71 ± 0.44	−3.75*	0.025
	Control	6.09 ± 0.69	6.20 ± 0.66	1.01	
HOMA-IR	Taekwondo	6.18 ± 6.27	2.07 ± 1.09	−66.53*	0.066
	Control	3.38 ± 2.20	3.95 ± 2.12	17.19	
GLP-1 (ng/ml)	Taekwondo	1.95 ± 0.50	2.80 ± 0.75	43.42**	<0.001
	Control	2.53 ± 1.66	1.78 ± 0.65	−16.82	

Values are represented as mean ± standard deviation.

Significantly different from baseline: *p < 0.05, **p < 0.01.

TC, total cholesterol; TG, triglyceride; HDL-C, high-density lipoprotein cholesterol; LDL-C, low-density lipoprotein cholesterol; FFA, free fatty acid; FBG, fasting blood glucose; HbA1c, percentage of glycosylated hemoglobin A1c; HOMA-IR, homeostatic model assessment for insulin resistance; GLP-1, glucagon-like peptide 1.

**Figure 3:**
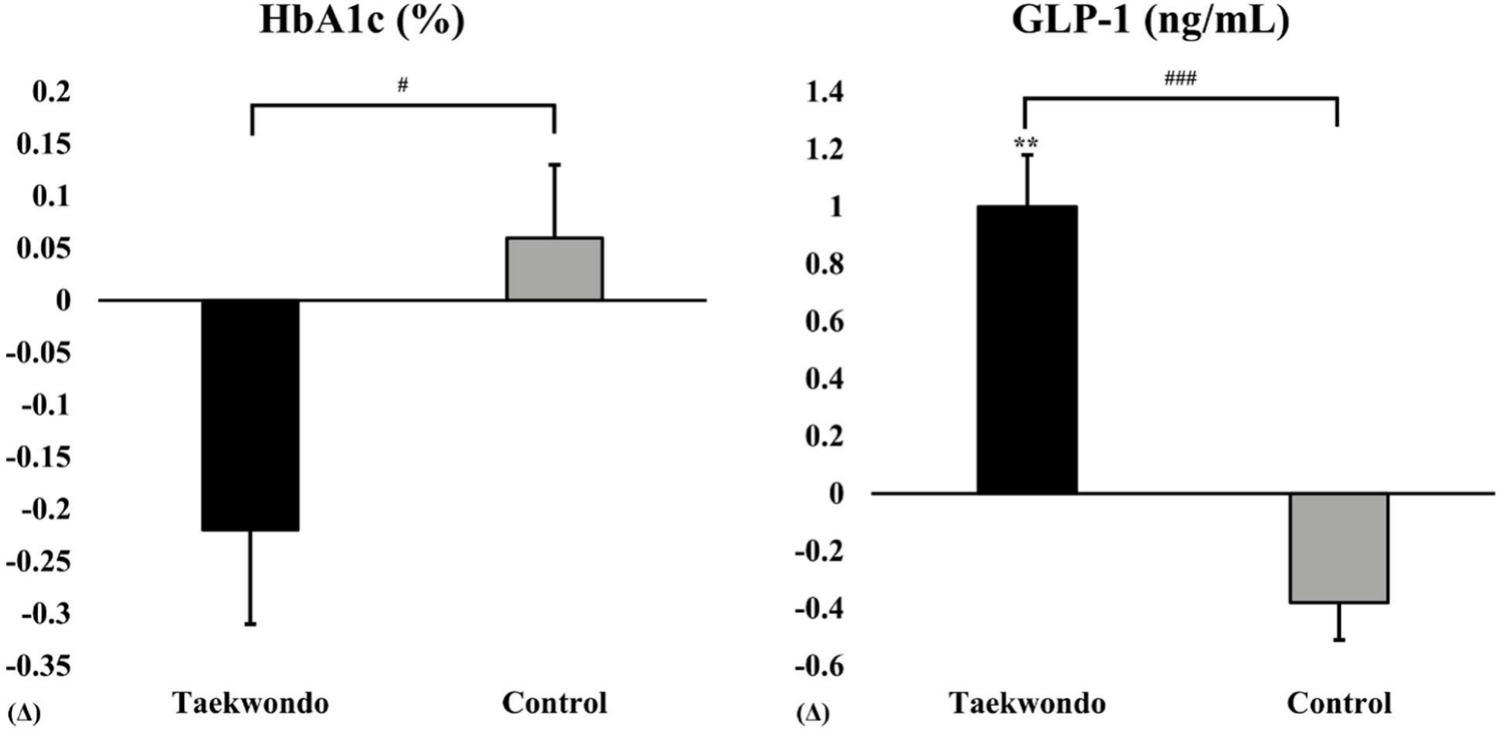
Comparison of HbA1c and GLP-1 between baseline and after 12 weeks in taekwondo program. #: *p*-value (interaction) #*p* < 0.05, ###*p* < 0.001. *: *p*-value (time) **p < 0.01. Δ: Post–Pre. HbA1c, percentage of glycosylated hemoglobin A1c; GLP-1, glucagon-like peptide 1.

As shown in Figure 3, HbA1c levels tended to decrease in the Taekwondo group compared to baseline, whereas a slight increase was observed in the control group. A significant interaction effect was found between the two groups (*p* < 0.05). Additionally, GLP-1 levels significantly increased in the Taekwondo group compared to baseline (*p* < 0.01), whereas a decreasing trend was observed in the control group. A significant interaction effect was observed between the two groups (*p* < 0.001).

### 3.4 Correlation between thigh muscle cross-sectional area and other variables

The thigh muscle CSA demonstrated significant negative correlations with TUG (*r* = −0.658, *p* < 0.01), TG (*r* = −0.638, *p* < 0.01), FFA (*r* = −0.506, *p* < 0.05), FBG (*r* = −0.535, *p* < 0.05), insulin (*r* = −0.615, *p* < 0.01), and HOMA-IR (*r* = −0.608, *p* < 0.01) (Table 6). In contrast, significant positive correlations were observed for LBM (*r* = 0.491, *p* < 0.05), HDL-C (*r* = 0.736, *p* < 0.01), and GLP-1 (*r* = 0.718, *p* < 0.01).

**Table 6:** Correlation coefficient between changes in thigh muscle cross-sectional area and other variables.

Variable	LBM (kg)	TUG (sec)	TG (mg/dl)	HDL-C (mg/dl)	FFA (μmol/L)
Thigh muscle cross-sectional area (mm^2^)	0.491*	−0.658**	−0.638**	0.736**	−0.506*
	FBG (pg/ml)	Insulin (μU/ml)	HOMA-IR	GLP-1 (ng/ml)	
	−0.535*	−0.615**	−0.608**	0.718**	

LBM, lean body mass; TUG, timed up-and-go test; TG, triglyceride; HDL-C, high-density lipoprotein cholesterol; FFA, free fatty acid; FBG, fasting blood glucose; HOMA-IR, homeostatic model assessment for insulin resistance; GLP-1, glucagon-like peptide-1.

* *p* < 0.05.

** *p* < 0.01.

## 4 Discussion

This study demonstrated that regular, long-term Taekwondo training significantly increased thigh muscle CSA in sedentary older women, an outcome of particular importance given the difficulty in improving muscle mass in this population. Increased thigh muscle CSA is associated with functional mobility, balance, and insulin sensitivity, thereby reducing fall risk and improving metabolic health, especially in older women with a sedentary lifestyle (22).

The significant improvements in thigh muscle CSA observed in this study may be due to the combined aerobic and resistance components of Taekwondo, particularly the dynamic kicking movements and postural changes that stimulate muscle growth (27, 47, 48). These movements effectively engage lower body muscles, promoting hypertrophy and strength in older adults. This is noteworthy as increasing muscle mass is challenging in older women due to age-related declines in muscle synthesis (49–53). However, Taekwondo's unique combination of dynamic resistance and functional movements appears particularly effective for stimulating muscle growth and improving insulin sensitivity in sedentary older women, highlighting its potential as a practical intervention for enhancing muscle mass and metabolic health in this population.

Increased thigh muscle CSA observed a positive association with the HRPF of sedentary older women. More specifically, the significant decline in the TUG test and the significant increase in single-leg stance test, maximum stride length in Taekwondo group indicate that Taekwondo training had a beneficial effects on balance and mobility in older individuals. These improvements are directly linked to the increases in thigh muscle CSA, highlighting the interconnectedness of muscle mass and physical function. The importance of regular exercise in sedentary older women are reducing the risk of falls, improving gait ability, and promoting independence in the older population (25, 54, 55). Our results indicate that the Taekwondo program has the potential to enhance HRPF, particularly balance and gait, thereby reducing fall risk and increasing mobility.

Additionally, significant reductions in HbA1c and increases in GLP-1 levels were observed, suggesting enhanced glucose metabolism. Given the role of GLP-1 in promoting insulin secretion and improving insulin resistance, the observed increase in GLP-1 provides a plausible mechanism for the improvements in HbA1c and insulin sensitivity (56). This highlights the potential of Taekwondo as a multifaceted intervention that not only enhances muscle mass and physical fitness but also positively impacts metabolic health.

These findings underscore the importance of incorporating Taekwondo training as a strategic intervention for enhancing thigh muscle CSA, improving HRPF, and promoting metabolic health in sedentary older women. However, several limitations should be considered. The study's small sample size and the lack of a follow-up limit the generalizability of the results. Future studies should include larger, more diverse populations and consider longitudinal designs to assess the sustainability of the observed benefits. Furthermore, exploring the molecular mechanisms underlying the improvements in muscle mass and metabolic health will provide deeper insights into the efficacy of Taekwondo as an intervention for older adults.

## 5 Conclusion

Regular, long-term Taekwondo training has been demonstrated to enhance HRPF, total muscle mass, and thigh muscle CSA in sedentary older women, thereby contributing to improved insulin resistance risk factors. These findings suggest that Taekwondo training may be an effective exercise program to promote metabolic health in this population.

## Data Availability

The raw data supporting the conclusions of this article will be made available by the authors, without undue reservation.
